# Supplementary figures and images for: Mechanisms of active diffusion of vesicular stomatitis virus inclusion bodies and cellular early endosomes in the cytoplasm of mammalian cells
Source: PLoS One. 2024 Mar 14;19(3):e0290672. doi: 10.1371/journal.pone.0290672 (PMC10939199; doi:10.1371/journal.pone.0290672)

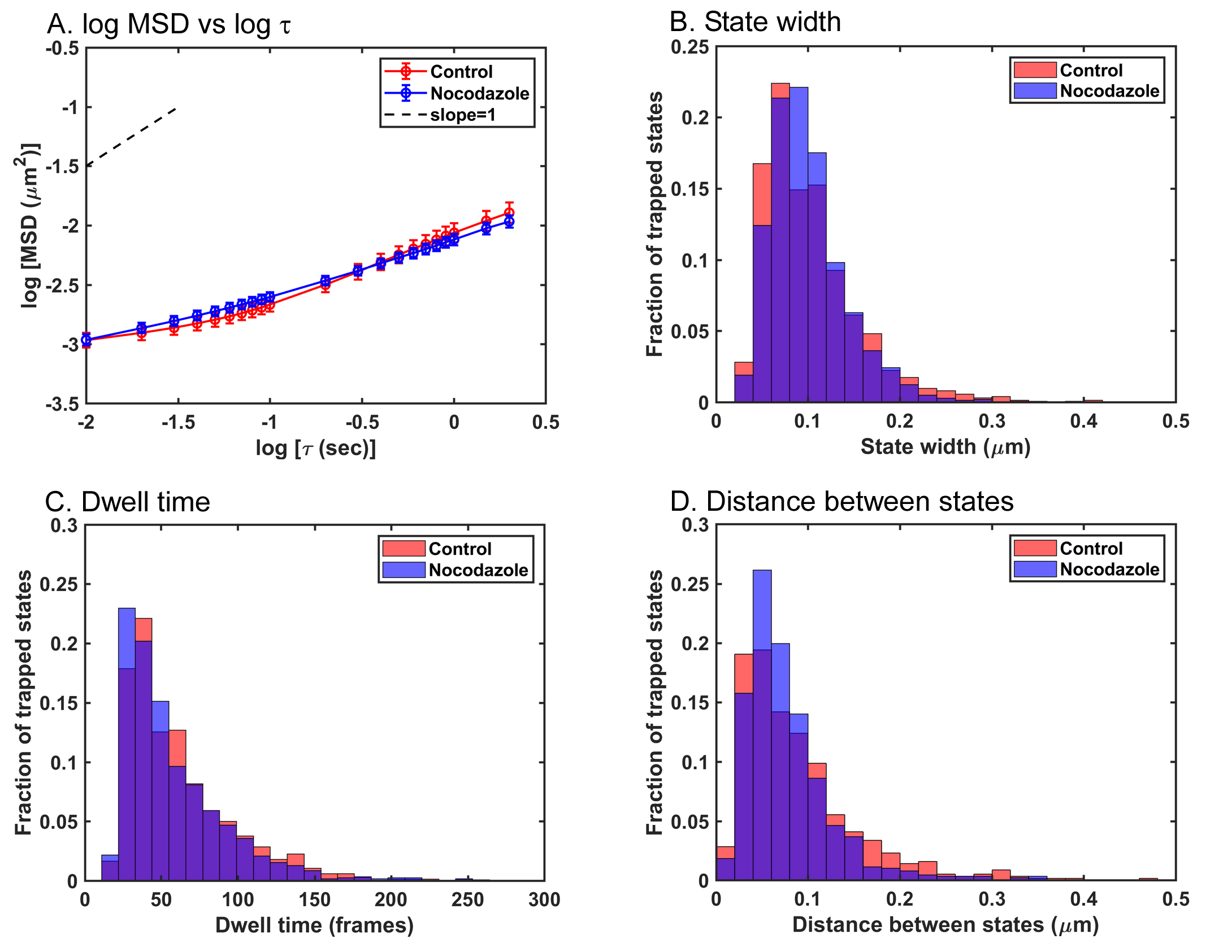

Supplement: S1 Fig — Mean squared displacements (MSDs) of early endosomes that only occupied trapped states were determined as a function of time delay (τ) and are shown as a log-log plot (A). The data shown are means ± 95% confidence intervals (n = 151 tracks from control cells and 238 from nocodazole-treated cells) obtained in three independent experiments. Variational Bayesian analysis was used to calculate the widths of trapped states occupied by early endosomes (B), dwell times of the early endosomes in those trapped states (C), and the distance between states (D). States in nocodazole-treated cells (n = 1353) were slightly larger than those in control cells (n = 1206), but the difference was not statistically significant (p > 0.05, Mann-Whitney test). The dwell times of early endosomes occupying trapped states in nocodazole-treated cells (n = 1149) were significantly shorter than those in control cells (n = 660) (p < 0.05, Mann-Whitney test). The distance between states was slightly shorter for early endosomes occupying trapped states in nocodazole-treated cells (n = 237 tracks) than the distance between states in control cells (n = 150 tracks) (p > 0.05, Mann-Whitney test). (TIF) [file pone.0290672.s001.tif]
