# Supplementary material for: Mechanisms of active diffusion of vesicular stomatitis virus inclusion bodies and cellular early endosomes in the cytoplasm of mammalian cells
Source: PLoS One. 2024 Mar 14;19(3):e0290672. doi: 10.1371/journal.pone.0290672 (PMC10939199; doi:10.1371/journal.pone.0290672)
Supplement: Attachment — Submitted filename: ResponseToReviewers.pdf [file pone.0290672.s003.pdf]

Response to Reviewers' Comments

We thank the reviewers for their thoughtful comments and have revised the manuscript in response as described here. Changes from the original version are indicated in red font in the marked revised version.

Reviewer 1:

1) "The movement[s] of trapped inclusion bodies and trapped early endosomes are very small . . . on the order of 100 nm . . . below optical resolution. This raises some questions about the significance of the observation."

Response: The Video Spot Tracker software uses data from multiple pixels to calculate the position of the center of the fluorescent particles to subpixel resolution, but the reviewer raises an important point. Localization error, which can arise from stage vibrations, and the subpixel tracking error generated by Video Spot Tracker, was determined from the localization error in the position of 1µm latex beads stuck to the surface of a cover slip to be 0.023 µm. Thus although the distances traveled from trap to trap are small relative to the size of the particles, they are at least 3- to 4-fold above the localization error. These points are included in lines 227-230 and 523-525 in the revised manuscript.

2) "Can the authors completely exclude that . . . the inclusion bodies are of smaller average size after treatment . . . [or] if the composition of the inclusion bodies is affected due to drug treatment."

Response: The sizes of the inclusion bodies that were analyzed in latrunculin A-treated cells were not significantly different from those in control cells. Histograms illustrating this point are included in the revised manuscript as Fig 1E, and the statistical results are described in lines 168-170 and in the legend for Fig 1E. Much more difficult to exclude is the possibility that drug treatment changes the composition of the inclusion bodies. This possible explanation for the results is included in lines 209-211.

Reviewer 2:

1) “Can the authors comment on the size distribution statistics and how many VSV inclusion bodies they analyze in Figure 1? A histogram . . . would be appropriate here. . . . The authors should validate whether the particles they track are similar in size.”

Response: This is similar to comment 2 of Reviewer 1. The addition of the histograms in Fig 1E and the description of the statistics in the revised manuscript are in response to both reviewers’ comments.

2) “The requirements for LLPS given in [the original manuscript] are misleading and incorrect. . . .Broadly the authors, who don’t require LLPS for the impact of their paper, should lessen or remove these claims of rules and instead cite the previous papers and move on.”

Response: This was an excellent suggestion, especially in light of comment 3. The indicated text was deleted in the revised manuscript.

3) “Most in the field prefer to avoid the prefix "liquid-liquid" . . . it probably isn't advisable given the contentious nature of LLPS (Musacchio EMBO 2022).”

Response: The reviewer’s and Professor Musacchio’s points are well-taken, and many claims of liquid-liquid phase separations (LLPS) could be due to alternate mechanisms, such as rapidly exchanging site-specific interactions. We have changed references to LLPS in the revised manuscript to the more general term “membraneless cellular compartments” as in the title of the referenced paper, which is also now cited in the revised text of the manuscript (lines 99-104).

4) “Line 115, "like our previous study," is missing a citation.”

Response: Thank you, citations added (now line 110).
